# Cerebral Astroblastoma: A Rare Tumor

**DOI:** 10.7759/cureus.16323

**Published:** 2021-07-12

**Authors:** Biswajit Dey, Satya Dutta, Animesh Saurabh, Vandana Raphael, Yookarin Khonglah

**Affiliations:** 1 Pathology, North Eastern Indira Gandhi Regional Institute of Health and Medical Sciences (NEIGRIHMS), Shillong, IND

**Keywords:** astroblastoma, squash, immunohistochemistry, grade, pseudorosette

## Abstract

Astroblastoma is a rare neuroepithelial tumor of the central nervous system, which accounts for only 0.45-2.8% of all neuroglial tumors. These tumors have distinct radiological, histopathological, immunohistochemical, and molecular features. We describe a case of astroblastoma of the left temporal lobe in a 38-year-old female, who presented with complaints of headache and occasional episodes of vomiting.

## Introduction

Astroblastoma is a rare neuroepithelial tumor of the central nervous system of uncertain origin. It accounts for only 0.45-2.8% of all neuroglial tumors [1,2]. It predominantly affects the cerebral hemispheres of children and young adults but may also involve other parts of the brain [1,2]. Histopathology, immunohistochemistry (IHC), and neuroimaging are all required for reaching a clear diagnosis of this rare neoplasm as it shares the same histological features with ependymoma [3]. The Consortium to Inform Molecular and Practical Approaches to CNS Tumor Taxonomy (cIMPACT-NOW) update 6 has described MN1 alteration in astroblastoma [4]. In this report, we describe the crush cytology, histopathology, and immunohistochemical features of astroblastoma in a 38-year-old female, who presented with complaints of headache and occasional episodes of vomiting.

## Case presentation

**Figure 1:**
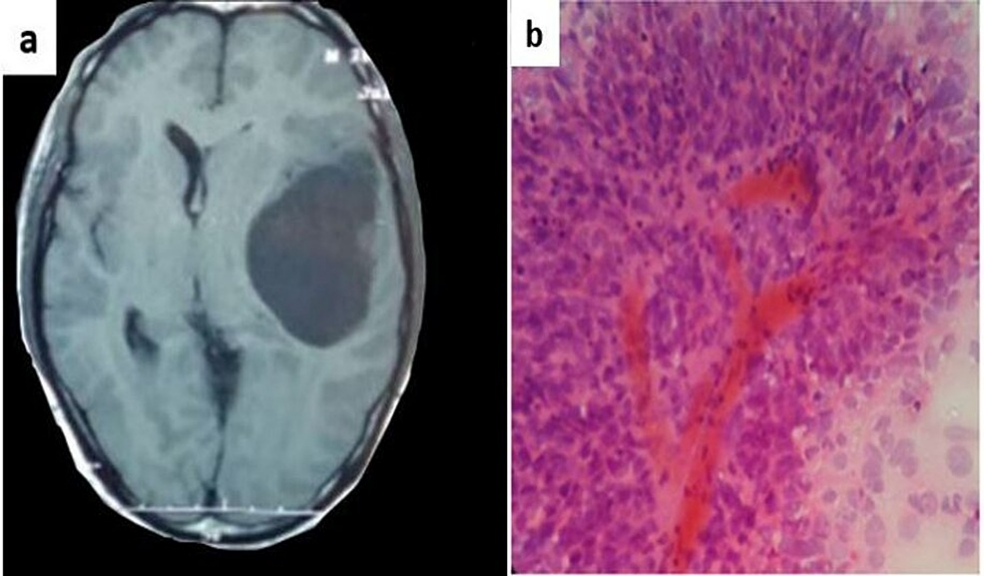
(a) CT showing a space-occupying lesion in the left temporal lobe. (b) Crush smear showing a papillary pattern in a fibrillary background with perivascular pseudorosettes (rapid H & E, 40x) H & E: hematoxylin and eosin

A 38-year-old female presented with complaints of headache and occasional episodes of vomiting for a duration of one month. There was no history of seizure or any other significant medical history. On physical examination, the patient was fully conscious and well oriented about time, place, and person with a Glasgow Coma Scale score of 15/15. There was no pupillary deficit or motor deficit. Other systemic examinations were within normal limits. CT scan of the brain showed a left temporal space-occupying lesion (SOL), which was cystic with heterogenous enhancing solid component suggestive of a glial neoplasm (Figure 1a). Left temporal craniotomy was done. Intraoperatively, a highly vascular circumscribed tumor was seen. Multiple grey-white to reddish soft tissue bits amounting to 1 gm were collected for intraoperative cytology consultation. Squash smears were made by crushing the unfixed 1-2 mm tissue bit between two slides. The smears were fixed immediately in 95% alcohol, and rapid hematoxylin and eosin stain (H & E) staining was done. The smears were highly cellular, comprised of papillary and pseudopapillary arrangements of cells in the background of fibrillary material. Perivascular pseudorosettes formation was also noted. These cells were oval to elongated, containing abundant eosinophilic cytoplasm with single, cytoplasmic processes extending to central blood vessels. These cytoplasmic processes that extended to central blood vessels were broad and tapering (Figure 1b). Mitosis was occasionally present. Correlating with clinical, radiological and, cytological features, the differential diagnoses of astroblastoma and ependymoma were suggested.

**Figure 2:**
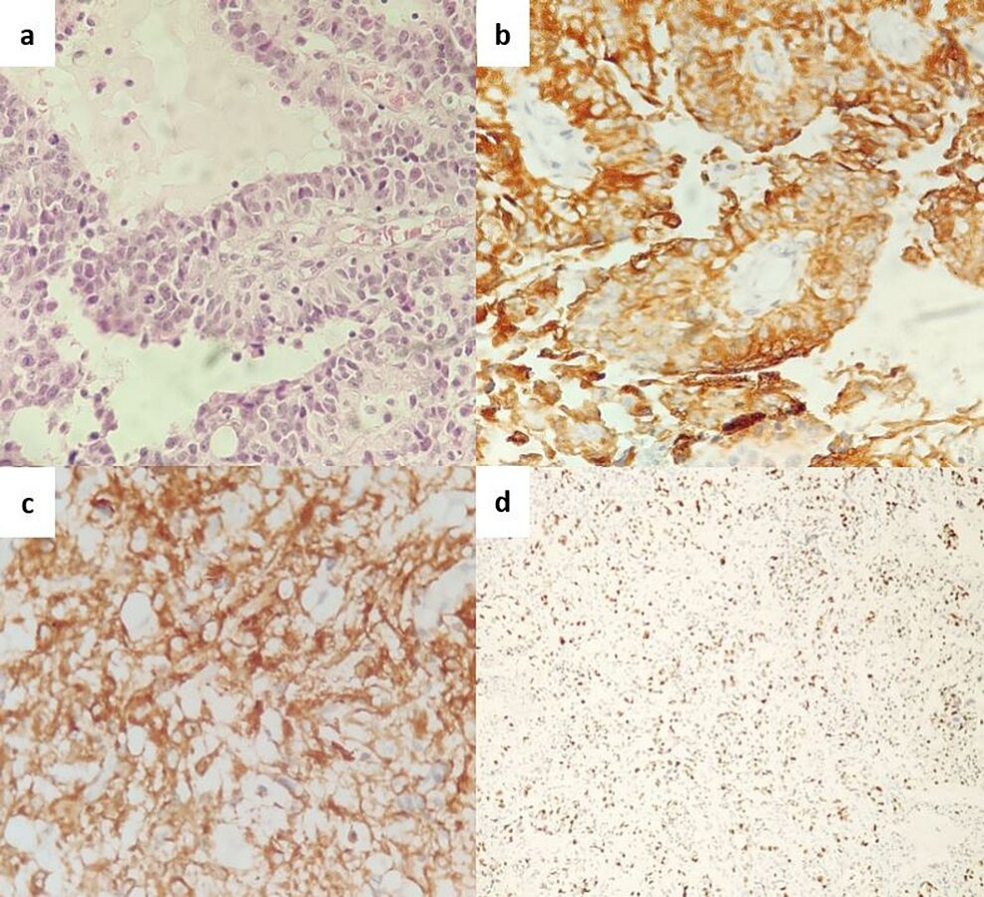
(a) Histopathology showing a papillary architecture and pseudorosettes around central hyalinized thickened blood vessels (H & E, x200). (b) Tumor cells positive for GFAP (IHC, x100). (c) Tumor cells positive for vimentin (IHC, x400). (d) Biopsy showing high Ki-67 index (IHC, x40) H & E: hematoxylin and eosin; IHC: immunohistochemistry; GFAP: glial fibrillary acidic protein

In view of differential diagnoses of astroblastoma and ependymoma in intraoperative cytology, a near-total excision of the tumor was done. Histopathological sections showed a cellular tumor comprising mostly of epithelioid-like cells with short and broad cytoplasmic processes arranged in perivascular pseudorosettes (Figure 2a). Perivascular fibrosclerosis and hyalinization were also seen. Focal areas of necrosis were noted. The mitotic rate was 6 per 10 high-power fields (HPF). Immunohistochemically, the tumor cells showed strong glial fibrillary acidic protein (GFAP), S100, and vimentin positivity (Figures 2b, 2c). Epithelial membrane antigen (EMA) showed membranous positivity. Pancytokeratin was focally positive. Isocitrate dehydrogenase 1 (IDH1) and L1 cell adhesion molecule (L1CAM) were negative. ATRX staining was retained. The p53 labeling index was <1%, and the Ki-67 index was 50% (Figure 2d). VE1 protein expression, which correlates with BRAFV600E gene mutation, was found to be negative on IHC.

A final diagnosis of high-grade astroblastoma was made. Since MN1 alteration could not be done, the diagnosis was made as astroblastoma, NOS (Not Otherwise Specified) as per the guidelines of cIMPACT-NOW update 6. The patient was given radiation therapy and a close follow-up was advised. He was found to be doing well after six months of follow-up.

## Discussion

An extremely rare tumor, astroblastoma was formerly designated as “neuroepithelial tumors of uncertain origin” [5]. Currently, the WHO classification of CNS tumors has classified astroblastomas in the category of “other neuroepithelial tumors” [5]. A bimodal age of presentation is commonly seen, between 10-30 years in most of the reported cases [6]. This is in contrast with other glial tumors that affect older adults, while ependymoma is commonly found in younger children. There is a female predominance among patients diagnosed with astroblastoma [3,7].

Cytologically, astroblastoma is a densely cellular tumor. The tumor cells are arranged around blood vessels showing a rosette-like arrangement forming pseudorosettes [8]. The histological differential diagnoses of astroblastoma are anaplastic astrocytoma, glioblastoma, and ependymoma. Histologically, astroblastoma is composed of a monotonous population of spindle-shaped cells with coarse chromatin. These cells are directed towards the blood vessel by a short and broad tapering process forming pseudorosettes [9]. Glioblastoma and anaplastic astrocytoma also show pseudorosette, but in these tumors, it is focal, while in astroblastoma, these are universally distributed all over the tumor tissue. In the ependymal pseudorosette, the cells are more compactly arranged around the blood vessels with thin and tapering cytoplasmic processes toward the central vessel [10].

These tumors show immunopositivity for GFAP, EMA, pancytokeratin, oligodendrocyte transcription factor 2 (OLIG2), vimentin, podoplanin, and S100, and are negative for IDH1/2 and TP53 mutations [11]. Vimentin and S100 are more characteristic of an astrocytic origin [3,10]. In the present case, the tumor was positive for GFAP, vimentin, and S100. EMA showed membranous positivity and pancytokeratin was focally positive. The membranous pattern of immunopositivity of EMA as opposed to the perinuclear dot pattern and negative immunoreactivity for L1CAM ruled out ependymoma in the present case. The negative expression for IDH1, retained ATRX, and p53 labeling index <1% ruled out diffuse glioma as well.

Differentiation of low-grade (well-differentiated) and high‑grade (malignant) tumors is important for the therapeutic purpose, and it is based on the presence of mitoses, Ki-67 index, degree of cellular pleomorphism, and area of necrosis. Low-grade astroblastomas mostly show areas of pseudorosette formations around blood vessels (astroblastoma pseudorosettes) with extensive sclerosis [8,11]. The high-grade tumor is highly cellular with anaplastic nuclear features, increased mitoses of >5 per 10 HPF, microvascular proliferation, and necrosis with palisading [8,11]. The Ki-67 proliferation index in these malignant astroblastomas is usually >10%. The high-grade tumor would warrant consideration of adjuvant radiotherapy in addition to resection [11]. In the present case, the tumor was graded as high-grade based on high cellularity with focal necrosis, mitotic rate >5 per 10 HPF, and Ki-67 proliferation index >10%.

It has been postulated that MN1 rearrangement with fusion partner genes, such as BEND2 and, less frequently, CXXC5, and other unidentiﬁed genes, may drive genetic alteration of astroblastomas [4]. The cIMPACT-NOW update 6 has defined astroblastoma with MN1 rearrangement as astroblastoma, MN1-altered [4]. Tumors showing classic astroblastoma morphology in which MN1 alterations cannot be tested could be labeled as ‘astroblastoma, NOS’ [4]. Histologically classic astroblastomas that have been tested but lack MN1 alterations and do not carry other molecular alterations could be designated as ‘astroblastoma, NEC (Not Elsewhere Classified)’ [4]. A subset of astroblastomas also harbors BRAFV600E mutation, which is also seen in ependymomas and in other high-grade gliomas like epithelioid glioblastoma and anaplastic pleomorphic xanthoastrocytoma [12,13]. However, the role of BRAFV600E mutation is not known in the pathogenesis of astroblastoma [4].

To support the diagnosis and prognosis of astroblastoma, imaging studies provide additional clues. T1- and T2‑prolongation relative to white matter with well‑demarcated boundaries and heterogeneous solid-cystic components with inhomogeneous contrast enhancement are often seen in astroblastoma [3,10]. Astroblastoma is supratentorial in location, whereas ependymoma can arise from posterior fossa in over 60% of cases and supratentorial compartment in 30% of cases [10]. Although peripheral rim enhancement around its cystic components may resemble that of a necrotic glioblastoma, minimal peritumoral white matter T2‑prolongation is usually seen in astroblastomas. Calcifications are commonly found, while it is unusual for glioblastoma [10].

## Conclusions

Astroblastomas are very rare neoplasms with distinct radiological and histopathological characteristics. Radiologically, astroblastomas are contrast-enhancing with both solid and cystic components. Histologically, they are well-characterized by astroblastic pseudorosettes and perivascular hyalinization. To rule out the various close differential diagnoses, ancillary techniques like IHC and cytogenetics are important. These tumors are positive for GFAP, EMA, pancytokeratin, Olig2, vimentin, podoplanin, and S100, and are negative for IDH1/2 and TP53 mutations. Astroblastomas reveal MN1 alteration, which has been implicated in their pathogenesis.

## References

[1] Eom KS, Kim JM, Kim TY (2008). A cerebral astroblastoma mimicking an extra-axial neoplasm. J Korean Neurosurg Soc.

[2] Agarwal V, Mally R, Palande DA, Velho V (2012). Cerebral astroblastoma: a case report and review of literature. Asian J Neurosurg.

[3] Bergkåsa M, Sundstrøm S, Gulati S, Torp SH (2011). Astroblastoma - a case report of a rare neuroepithelial tumor with complete remission after chemotherapy. Clin Neuropathol.

[4] Louis DN, Wesseling P, Aldape K (2020). cIMPACT-NOW update 6: new entity and diagnostic principle recommendations of the cIMPACT-Utrecht meeting on future CNS tumor classification and grading. Brain Pathol.

[5] Samples DC, Henry J, Yu FF, Bazan C, Tarasiewicz I (2016). A case of astroblastoma: radiological and histopathological characteristics and a review of current treatment options. Surg Neurol Int.

[6] Shen F, Chen LC, Yao Y, Zhou LF (2014). Astroblastoma: rare incidence and challenges in the pattern of care. World Neurosurg.

[7] Singh DK, Singh N, Singh R, Husain N (2014). Cerebral astroblastoma: a radiopathological diagnosis. J Pediatr Neurosci.

[8] Hammas N, Senhaji N, Alaoui Lamrani MY, Bennis S, Chaoui EM, El Fatemi H, Chbani L (2018). Astroblastoma - a rare and challenging tumor: a case report and review of the literature. J Med Case Rep.

[9] Fu YJ, Taniguchi Y, Takeuchi S (2013). Cerebral astroblastoma in an adult: an immunohistochemical, ultrastructural and genetic study. Neuropathology.

[10] Bell JW, Osborn AG, Salzman KL, Blaser SI, Jones BV, Chin SS (2007). Neuroradiologic characteristics of astroblastoma. Neuroradiology.

[11] Hirose T, Nobusawa S, Sugiyama K (2018). Astroblastoma: a distinct tumor entity characterized by alterations of the X chromosome and MN1 rearrangement. Brain Pathol.

[12] Pradhan P, Dey B, Srinivas BH, Jacob SE, Rathakrishnan RK (2018). Clinico-histomorphological and immunohistochemical profile of anaplastic pleomorphic xanthoastrocytoma: report of five cases and review of literature. Int J Hematol Oncol Stem Cell Res.

[13] Lehman NL, Hattab EM, Mobley BC (2017). Morphological and molecular features of astroblastoma, including BRAFV600E mutations, suggest an ontological relationship to other cortical-based gliomas of children and young adults. Neuro Oncol.

